# Antimicrobial Susceptibility Profiles of *Salmonella* spp. Isolates from Clinical Cases of Waterfowl in Hungary Between 2022 and 2023

**DOI:** 10.3390/microorganisms12122462

**Published:** 2024-11-29

**Authors:** Ádám Kerek, Ábel Szabó, Ákos Jerzsele

**Affiliations:** 1 Department of Pharmacology and Toxicology, University of Veterinary Medicine, István utca 2, 1078 Budapest, Hungary; szabo.abel@student.univet.hu (Á.S.); jerzsele.akos@univet.hu (Á.J.); 2 National Laboratory of Infectious Animal Diseases, Antimicrobial Resistance, Veterinary Public Health and Food Chain Safety, University of Veterinary Medicine, 1078 Budapest, Hungary

**Keywords:** *Salmonella*, antimicrobial resistance, minimum inhibitory concentration, MIC, waterfowl, geese, ducks

## Abstract

The global spread of antimicrobial resistance is one of the most significant challenges of the 21st century. The waterfowl sector is an economically decisive part of the poultry industry, yet it remains under-researched, and its antibiotic usage is less monitored. Our study aimed to determine the antimicrobial susceptibility of avian pathogenic *Salmonella* strains, which are still prevalent in ducks and geese, against antibiotics critical for both animal and human health, and to compare these findings with human resistance data. We analyzed 71 *Salmonella* strains, collected by the National Reference Laboratory from samples originating from 29 settlements across Hungary between 2022 and 2023, using the minimum inhibitory concentration (MIC) method. Notably, the duck strains (*n* = 52) exhibited 57.7% resistance to potentiated sulfonamides, 28.8% resistance to doxycycline, and 25% resistance to cefotaxime. Among the geese strains (*n* = 19), 52.6% showed resistance to potentiated sulfonamides, followed by 26.3% resistance to doxycycline and amoxicillin–clavulanic acid, and 15.8% resistance to cefotaxime, ceftiofur, and ceftriaxone. When compared to human resistance data, we found significantly lower resistance levels for amoxicillin in ducks (20.0%) and geese (8.3%) in the Dél-Alföld region, compared to ampicillin resistance in human samples (45.4%), in which amoxicillin analog is an antibiotic in human medicine. Resistance to ciprofloxacin was only observed in ducks (2.0%), whereas pefloxacin resistance in human medicine was notably higher (22.3%). Overall, the results for the waterfowl sector in the Dél-Alföld region of Hungary align with the international literature in several aspects. Further investigation using next-generation sequencing to identify the genetic basis of multi-resistant strains is warranted.

## 1. Introduction

Antimicrobial resistance (AMR), sometimes referred to as a “silent pandemic” [1], is one of the most significant health concerns of our time and is a serious cause for concern worldwide. Indeed, it is now one of the 10 most common health risks in the world [2]. It is mainly the result of inappropriate antibiotic use, which is rising at an alarming rate [3]. The overuse of antibiotics is particularly serious in the agriculture of developing countries [4], with India being one of the biggest users of antibiotics, particularly in agriculture [5]. Despite the widespread use of antibiotics in certain regions, nearly 2 billion people around the world still lack access to essential medicines, including antibiotics, which are vital for treating common infectious diseases [6]. The lack of access to first- and second-line antibiotic therapies in developing countries often leads to the use of less effective or broader-spectrum agents, which further contribute to the development of AMR [7]. Recent studies [8,9,10], based on the profiling of antimicrobial resistance genes (ARGs) in soil and water, have shown that AMR is widespread not only in animals but also in the environment and that wild birds may be reservoirs [11].

As a result, the drive for prudent and responsible use of antibiotics is also growing. Regulations and legislation are being introduced worldwide to promote more rational use of antibiotics [12]. The European Medicines Agency’s (EMA) Ad Hoc Expert Group on Antimicrobials (AMEG) classifies antibiotics used in animals into four broad groups, considering their impact on resistance in public health in 2019. Active substances that should be avoided were classified in category “AMEG A” (Avoid), those that should be restricted in category “AMEG B” (Restrict), those that should be used with caution in category “AMEG C” (Caution), and those that should be used with prudence as first-line agents in category “AMEG D” (Prudence) [13]. Additionally, the application of therapy based on appropriate pharmacokinetic/pharmacodynamic models is essential [14]. AMR is especially important in food-borne infections such as septicemia resulting from infection with *Salmonella* species [15]. To avoid further escalation of resistance of these bacteria, efforts should also be made to replace antibiotic use in agriculture with alternatives such as antimicrobial peptides [16], various essential oils [17], plant extracts [18,19], or even propolis [20,21,22], particularly in the pig [23] and poultry [24] sectors, which are the biggest users of antibiotics.

In 2022, Hungary kept 2,727,000 ducks and 614,000 geese, accounting for 7.79% and 1.76% of the global duck and goose population, respectively [25]. The majority of these birds were exported. Despite Hungary’s success in implementing *Salmonella* eradication programs in the poultry sector, these efforts have not been extended to the waterfowl sector, which continues to pose a significant risk for infection. The risk of *Salmonella*-mediated infection of waterfowl is highlighted by a study of retail duck meat in southern China from 2009 to 2016, which found a *Salmonella* prevalence of 15.94%, with *Salmonella* Derby (28.48%) being the most common serotype. Regarding their phenotypic resistance profile, 63% of the strains were resistant to tetracyclines, and the prevalence of multidrug-resistant (MDR) strains has shown a continuous increase worldwide from the past few decades to the present day [26].

*Salmonella* spp. are Gram-negative bacteria belonging to the family Enterobacteriaceae. Of these, the enterica subspecies is primarily responsible for *Salmonella* infections in humans and animals. The Gallinarum and Pullorum serotypes cause infections in waterfowl, pigeons, sheep, and pigs [27]. The presence of *Salmonella* is a result of poor hygiene and indicates exposure to contaminated food or water [28]. The Gallinarum and Pullorum serotypes can be spread through water, milk, raw vegetables, seafood, and contaminated eggs, among other things [29].

Salmonellosis is the third leading cause of death among foodborne diseases in humans (FTD) [30]. It is considered one of the most important zoonotic pathogens in humans and is causing an estimated 93.8 million cases of gastroenteritis worldwide each year. About 9% of cases result from direct contact with animals. For human infections caused by *Salmonella* spp. in 2021, the European Food Safety Authority (EFSA) reported resistance levels of 47.9% for ampicillin, 3–5% for third-generation cephalosporins, 1.5% for gentamicin, and 14.5% for ciprofloxacin [31]. 

The purpose of the present study is to assess the antimicrobial resistance profile of *Salmonella* strains isolated from clinical cases in duck and goose flocks in Hungary and to compare the results with the resistance status of strains isolated from human hospital cases. The *Salmonella* control programs have not been extended to the waterfowl sector, resulting in a significant number of cases causing clinical disease and mortality. Poor hygiene practices or inadequate kitchen techniques contribute to the prevalence of *Salmonella* infections associated with this sector. Therefore, it is of paramount importance to monitor and track the antibiotic susceptibility of strains at the population level continuously. Resistance, particularly when the responsible resistance genes are located on plasmids, phages, or other mobile genetic elements, can easily be transferred to other bacterial species. Moreover, the increasing emergence of multidrug-resistant strains poses a severe risk by leading to life-threatening nosocomial infections due to the diminished efficacy of previously successful antibiotic treatments. In this context, the roles of veterinarians, human healthcare professionals, and researchers are crucial for monitoring and addressing these challenges through the lens of the One Health approach.

## 2. Materials and Methods

### 2.1. Origin of the Strains and Human Data

The 71 strains were isolated from clinical cases by the National Food Chain Safety Office, Veterinary Diagnostic Directorate, between February 2022 and May 2023, which acts as a National Reference Laboratory, and the identification of strains has been carried out according to a standard. In the National Reference Laboratory, the ISO 6579-1:2017 standard is applied [32]. Additionally, during the accreditation process, laboratory-specific methods are validated according to the requirements of the current Validation of Microbiological Methods standard (MSZ EN ISO 16140) [33]. Samples from suspected *Salmonella* cases were pre-enriched in buffered peptone water at 37 °C for 18–24 h. They were then incubated in Rappaport–Vassiliadis broth (Biolab Zrt., Budapest, Hungary) at 41.5 °C for 24 h, followed by incubation on Xylose Lysine Deoxycholate agar (Biolab Zrt., Budapest, Hungary) at 37 °C for 24 h. Colonies exhibiting typical morphology (red colonies with black centers) were transferred to non-selective agar and subjected to biochemical tests for *Salmonella* identification. These included Triple Sugar Iron (Biolab Zrt., Budapest, Hungary) and Lysine Iron Agar (Biolab Zrt., Budapest, Hungary), where gas production, glucose fermentation, and the absence of lactose fermentation confirmed the species. No serological testing was performed. All strains we tested were submitted for formal diagnostic testing, so the distribution of samples was random. The species identification of the strains was determined using MALDI-TOF mass spectrometry (Flextra-LAB Kft., Budapest, Hungary) and Biotyper software version 12.0 (Bruker Daltonics GmbH, Bremen, Germany, 2024) [34]. The cultures were frozen in a Microbank™ system (Pro-Lab Diagnostics, Richmond Hill, ON, Canada) at −80 °C. Human resistance data (2021–2023) were provided by the National Centre for Public Health and Pharmacy.

For each strain, the species (duck, goose), the organ (brain chamber, bone marrow, liver, oviduct, pericardium, lung), and the location from which the sample arrived were recorded. They were then classified into seven administrative regions of Hungary during the registration process based on their origin. This regional categorization allowed comparison with human resistance data using a bar chart. The aim of the comparison is to look for correlations and tendencies, thus striving for the One Health concept.

### 2.2. Determination of the Minimum Inhibitory Concentration (MIC)

The phenotypic expression of AMR was assessed by determining the minimum inhibitory concentration (MIC) values of each bacterial strain according to the Clinical Laboratory Standard Institute (CLSI) methodology [35,36]. The breakpoints were also defined according to CLSI guidelines [35,36], and compared with the epidemiological cut-off value (ECOFF) set by the European Committee on Antimicrobial Susceptibility Testing (EUCAST).

Bacterial strains stored at −80 °C were suspended in 3 mL of cation-adjusted Müller-Hinton broth (CAMHB) the day before analysis and incubated for 18–24 h at 37 °C. The investigations were carried out using 96-well microtiter plates (VWR International, LLC., Debrecen, Hungary). All wells except the first column of the working plates were filled with 90 µL CAMHB. A 1024 µg/mL stock solution of the test substances (Merck KGaA, Darmstadt, Germany) was prepared according to CLSI guidelines [35,36]. The active ingredients amoxicillin and amoxicillin–clavulanic acid in a 2:1 ratio (pH 7.2, 0.01 mol/L) and imipenem (pH 6, 0.1 mol/L) were dissolved in a phosphate buffer solution. Ceftiofur, cefotaxime, ceftriaxone, doxycycline, minocycline, neomycin, spectinomycin, tylosin, tiamulin, lincomycin, colistin and vancomycin were dissolved in distilled water. For the preparation of the potentiated sulfonamide (trimethoprim and sulfamethoxazole at a 1:19 ratio), sulfamethoxazole was dissolved in hot water with a few drops of 2.5 mol/L NaOH, while trimethoprim was dissolved in distilled water with 0.05 mol/L HCl. Enrofloxacin, ciprofloxacin, and levofloxacin were prepared using a few drops of 1 mol/L NaOH solution in distilled water. Florfenicol, chloramphenicol, and azithromycin were dissolved using a few drops of 95% ethanol and distilled water.

Then, 180 µL of a 512 µg/mL solution diluted half and half with broth was spiked into the first column of the working plates and used to prepare a 2-fold dilution series. After column 10 of the microtiter plate, the excess 90 µL of solution was discarded, leaving 90 µL of solution in each column. Using a nephelometer (ThermoFisher Scientific, Budapest, Hungary), a bacterial suspension set at 0.5 McFarland was inoculated backward from column 11 of the microtiter plates at 10 µL/well [35]. The evaluation was performed using the Sensititre™ SWIN™ automatic MIC reader (ThermoFisher Scientific, Budapest, Hungary) and VIZION system software version 3.4 (ThermoFisher Scientific, Budapest, Hungary, 2024). The reference isolate was *Salmonella enterica* (ATCC 35664).

### 2.3. Statistical Analysis

Statistical analysis of the data was performed using R program version 4.1.0 [37]. Normal distribution was tested using the Shapiro–Wilk test. Data not following normal distribution were further tested using non-parametric tests. The Kruskal–Wallis test was used to analyze the differences in the degree of resistance of each drug according to different criteria. This test does not assume a normal distribution of the data and can be used to compare the median of several sample groups, making it ideal for analyzing differences between groups. During the analysis, we determined which MIC values were classified as sensitive or resistant based on clinical breakpoints. The correlation analyses were conducted using continuous data.

We then applied an additional post hoc test to determine the exact correlations between groups. To do this, we used a Mann–Whitney U test, pairwise comparing each type, and then corrected for inflated *p*-values resulting from multiple comparisons using the Bonferroni correction. It should be noted that applying the Bonferroni correction may increase the chance of a type II error (failure to detect true differences). For each active substance, a chi-square test was performed to determine whether there was a significant difference in the level of resistance between the two species. When conducting correlation analyses, metadata were assigned based on specific criteria to determine correlations between antibiotic agents. Separate analyses were performed considering the isolation site and the exact species.

In data analysis, clusters refer to groups in which data show similar patterns or characteristics. This type of analysis can be useful for antibiotic selection and treatment protocols, especially when the aim is to better understand resistance patterns and develop more effective treatment strategies. The Random Forest machine learning algorithm, built with 100 decision trees, was used to assess the importance of data features. The classification report’s precision, recall, and F1-score values are shown for each cluster. During the analysis, the model accounted for all metadata.

## 3. Results

### 3.1. The Distribution and Regional Origin of the Samples Received

**Figure 1:**
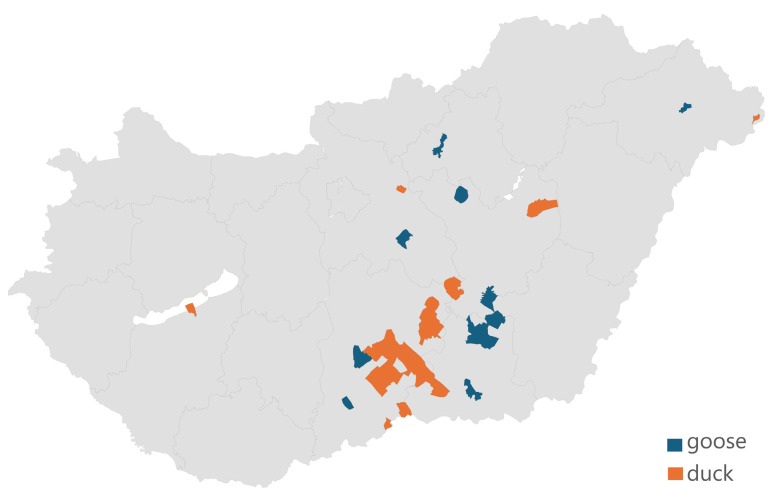
The geographical origin of the 71 *Salmonella* isolates received shows that 87.3% of the samples originated from the Dél-Alföld region, reflecting the concentration of the waterfowl industry in Hungary.

In total, 71 strains were analyzed. The distribution of samples by origin is summarized in Figure 1. Samples were received from a total of 29 different settlements, with the vast majority concentrated in the southern part of the country. A total of 69% of the samples came from ducks and 31% from geese. The results of species identification using the MALDI-TOF device are presented in Supplementary Tables S1–S4. The results showed a perfect match for all *Salmonella* sp. isolates, as we observed a logarithmic score bigger than two in all positive samples.

### 3.2. Antimicrobial Susceptibility Testing

A total of 52 isolates from duck samples were tested for MIC values against 21 different antibiotic agents. The active substances were selected based on their relevance to animal and public health and, in the case of the CLSI guideline, the breakpoints for *Salmonella* species. An additional selection criterion was to cover the widest possible range of antibiotic classes and to include active substances commonly used in both practice and research.

**Figure 2:**
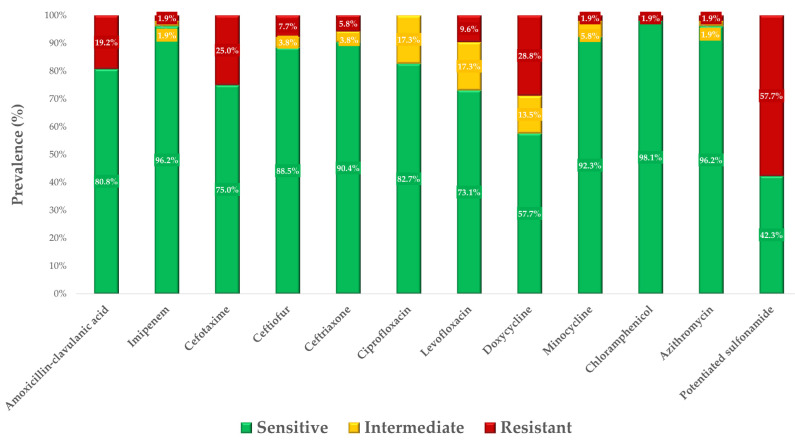
Antibiotic susceptibility profiles of *Salmonella* strains isolated from ducks (*n* = 52) to active substances of animal and public health importance showed that most critically important drugs retained their susceptibility. However, higher levels of resistance were observed for doxycycline and potentiated sulfonamide (trimethoprim–sulfamethoxazole 1:19).

Breakpoints were available for 10 of the 21 active substances, and the sensitivity profiles of the strains are illustrated in Figure 2. The corresponding exact MIC values are presented in Supplementary Table S1. For the other nine active substances, no breakpoints are available, and, therefore, the MIC values are summarized in Supplementary Table S2, including their MIC_50_ and MIC_90_ values. All data are included in the Additional data file.

The highest rate of resistance was observed with potentiated sulfonamide, followed by doxycycline, and cefotaxime. The best situation was observed with ciprofloxacin, where 82.7% of strains were sensitive and 17.3% were intermediate. For chloramphenicol, 98.1% of strains were found to be sensitive. 

**Figure 3:**
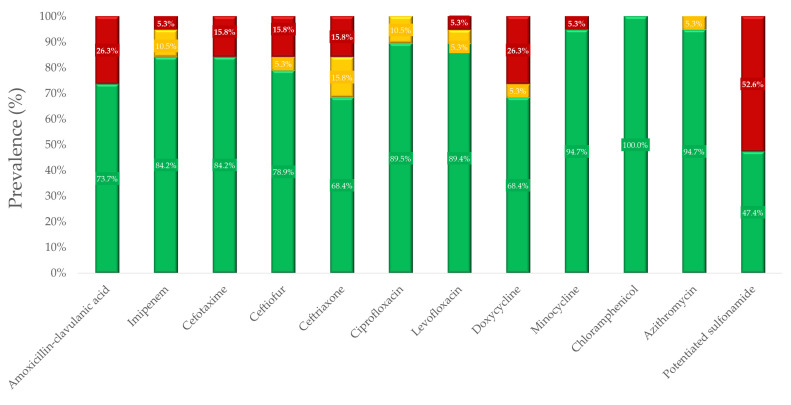
Antibiotic susceptibility profile of *Salmonella* strains isolated from geese shows that doxycycline and potentiated sulfonamide (trimethoprim–sulfamethoxazole 1:19) exhibited the highest levels of resistance.

For geese samples, a total of 19 *Salmonella* isolates were tested for susceptibility to the same active substances as in ducks. The sensitivity profile calculated from the breakpoints defined by the CLSI guidelines is illustrated in Figure 3. The MIC values for these results are summarized in Supplementary Table S3. The MIC values for active substances without breakpoints, along with the calculated MIC_50_ and MIC_90_ values, are provided in Supplementary Table S4.

In geese samples, the highest resistance was observed with potentiated sulfonamide (52.6%), followed by doxycycline (26.3%), and amoxicillin–clavulanic acid (26.3%). Resistance to cephalosporins (cefotaxime, ceftiofur, and ceftriaxone) was at a similar level of 15.8%. The best situation was with chloramphenicol, where strains showed 100% sensitivity.

The results showed no significant difference (*p*-value > 0.05) in the level of resistance between ducks and geese for any of the active substances (Supplementary Table S5).

**Figure 4:**
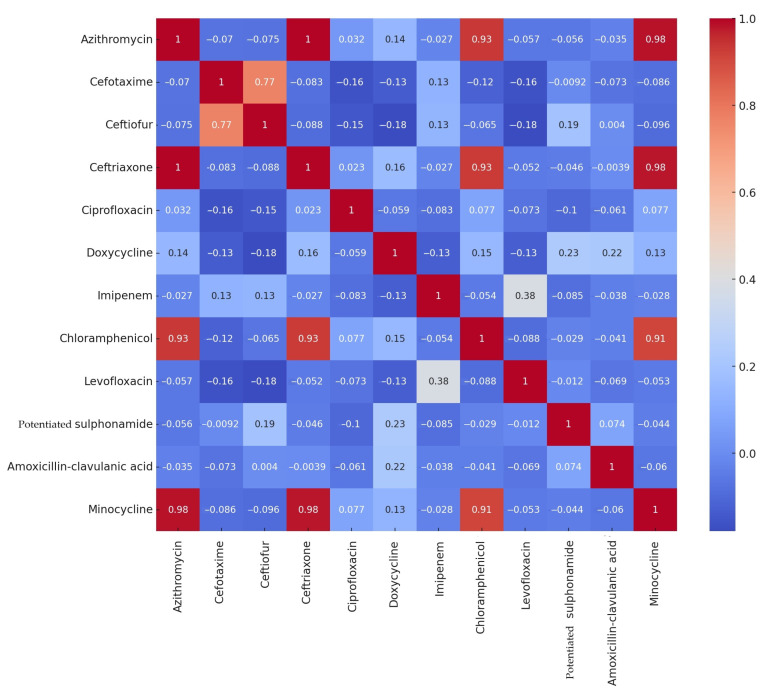
Correlation patterns between antimicrobial resistance patterns of *Salmonella* samples isolated from ducks per active substance. Values closer to 1 show a strong positive correlation (e.g., azithromycin and ceftriaxone), whereas negative values indicate opposite resistance patterns. Values close to zero suggest that there is no relationship between the resistance patterns of the compared substances.

**Figure 5:**
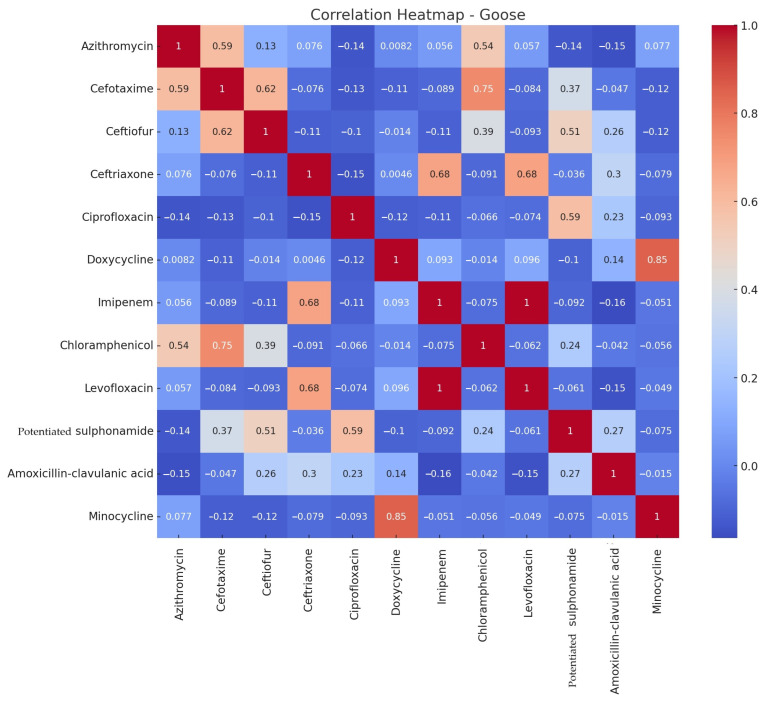
Correlation patterns between antimicrobial resistance patterns of *Salmonella* samples isolated from geese by active substance. Values closer to 1 show a strong positive correlation (e.g., doxycycline and minocycline), whereas negative values indicate opposite resistance patterns. Values close to zero suggest that there is no relationship between the resistance patterns of the compared substances.

The correlation analysis examined how the MIC values of each antibiotic substance correlate with each other. This was performed separately for ducks (Figure 4) and geese (Figure 5). The correlation is positive (+1) if the antibiotic resistance patterns are similar, negative (−1) if the patterns of antibiotic resistance are opposite, and close to 0 if there is no relationship between antibiotic resistance patterns.

In ducks, there was a strong positive correlation between azithromycin–ceftriaxone, azithromycin–chloramphenicol, azithromycin–minocycline, ceftriaxone–chloramphenicol, ceftriaxone–minocycline, and a significant correlation between ceftiofur–cefotaxime. In geese, weaker positive correlations were observed, with notable correlations between doxycycline–minocycline and cefotaxime–chloramphenicol.

**Figure 6:**
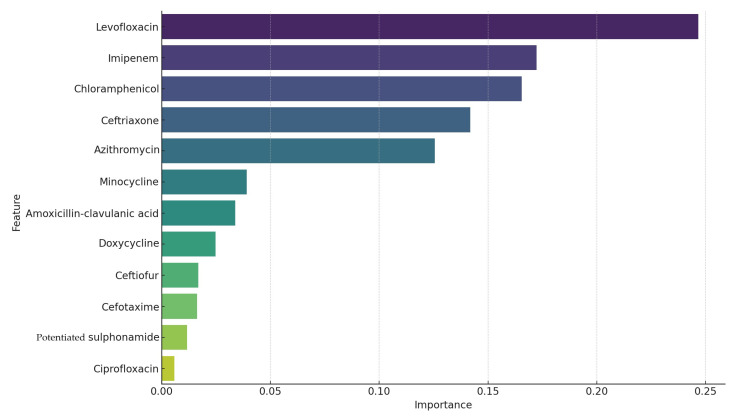
The analysis of the resistance profile of isolated samples per drug using the *Random Forest* method. “Importance” values are numbers between 0 and 1 that indicate how much a given characteristic contributed to the performance of the model. This is calculated by examining how much a given feature improves the performance of decision trees in discriminating between clusters. For each tree, the performance improvements caused by a given trait are summed and normalized so that the importance of all traits sums to 1.

The accuracy of the model was 0.95 (95%), meaning that the Random Forest model (Figure 6) correctly classified 95% of the samples into clusters. The high accuracy indicates that the model performed well in identifying differences between clusters of bacterial strains based on antibiotic resistance patterns. These antibiotic resistance patterns played the largest role in identifying different clusters of bacterial strains. 

In this case, of the five highlighted drugs, levofloxacin was the most important feature, contributing the most to model performance, while azithromycin was less important but still played a significant role in distinguishing the clusters. The order of importance of antibiotics in the model was: levofloxacin, imipenem, chloramphenicol, ceftriaxone, and azithromycin.

The degree of resistance in correlation with the different organs was also examined. We were curious to see if there was a relationship between resistance rates by antibiotics, assigning the organ of isolation from the metadata. There was a strong positive association between azithromycin–ceftriaxone (0.76), azithromycin–doxycycline (0.65), chloramphenicol–minocycline (0.99), and amoxicillin–clavulanic acid–doxycycline (0.98). However, there was a strong negative association between ciprofloxacin–ceftriaxone (−0.40). This noticeable negative correlation suggests that a high resistance rate for one antibiotic may be associated with a low resistance rate for the other antibiotic in different organs. This analysis helps to understand the relationships between bacterial isolation organs and antibiotic resistance patterns and can be beneficial for clinical decision guidance.

We investigated the correlation between the resistance pattern of each agent and the *Salmonella* species presumed to have the highest log score using the MALDI-TOF (Flextra-LAB Kft., Budapest, Hungary) and Biotyper software version 12.0 (Bruker Daltonics GmbH, Bremen, Germany, 2024). This study shows a strong positive correlation between azithromycin–ceftriaxone (0.99), chloramphenicol–azithromycin (0.97), amoxicillin–clavulanic acid-azithromycin (0.91), and ciprofloxacin–doxycycline (0.89). There was a strong negative correlation between ciprofloxacin–cefotaxime (−0.31) and ciprofloxacin–potentiated sulfonamide (−0.31).

**Figure 7:**
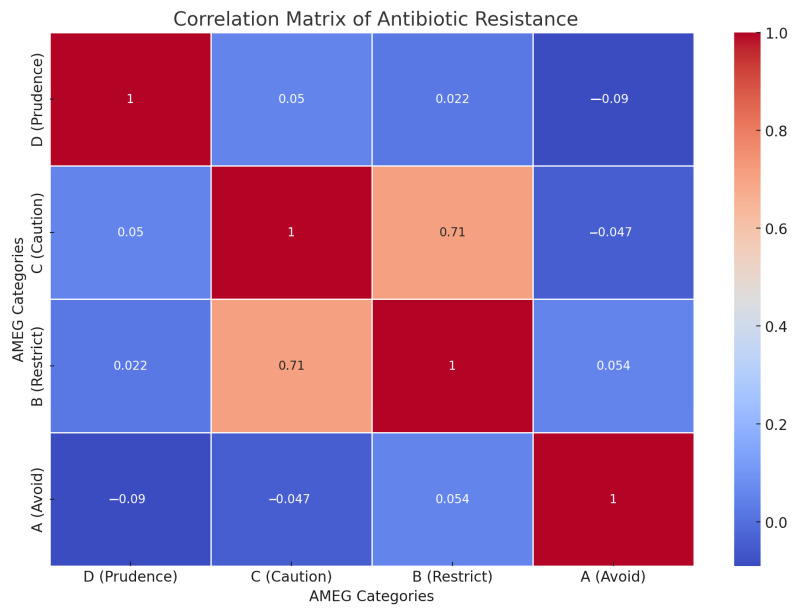
Correlation study following AMEG categorization of tested antibiotic active substances of animal and public health importance. A strong positive correlation was observed between categories C (Caution) and B (Restrict) (0.71).

The correlation matrix in Figure 7 shows the relationship between the level of antibiotic resistance of the test agents classified in each AMEG category (A, B, C, D). There is a strong positive correlation between categories C (Caution) and B (Restrict) (0.71), indicating that the resistance patterns of antibiotics in these categories are similar. There are weak negative correlations between categories D (Prudence) and A (Avoid) (−0.09) and between categories C (Caution) and A (Avoid) (−0.05).

These weak negative correlations indicate that the resistance patterns in the respective categories are slightly inversely related. This analysis can contribute to a better understanding of antibiotic resistance patterns according to AMEG categories and can be useful for clinical decision-making and antibiotic selection.

Table 1 summarizes the prevalence profile of the duck samples tested for the active substances for which breakpoints could be determined based on the CLSI guidelines. From these values, MIC_50_ and MIC_90_ were calculated, and the epidemiological cut-off value (ECOFF) values established by EUCAST were added. MIC_50_ and MIC_90_ values were both below the ECOFF values only for azithromycin. In terms of breakpoints, the MIC_50_ value was higher for doxycycline and potent sulfonamide, indicating that 50% of the study population is considered resistant to these agents. For MIC_90_ values, only azithromycin showed that 90% of the population was sensitive.

Table 2 shows the same results for the goose strains. In this case, 50% of the population was observed to be resistant only to the potent sulfonamide. When MIC_90_ values were monitored, 90% of the study population remained sensitive to azithromycin, ceftriaxone, ciprofloxacin, imipenem, chloramphenicol, and levofloxacin.

**Figure 8:**
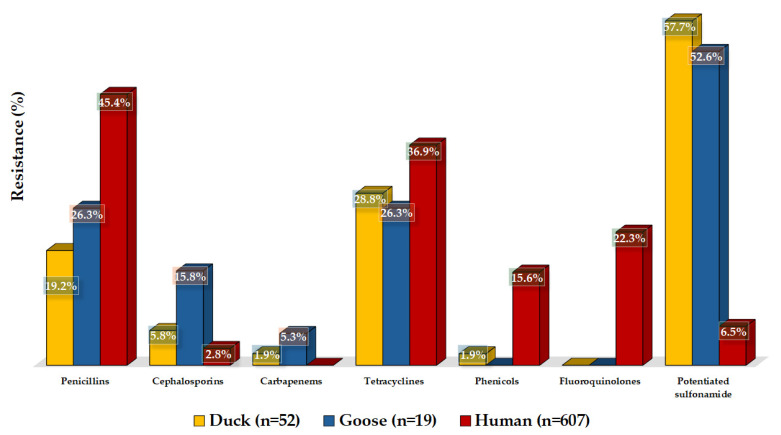
Resistance profile of duck and goose isolates tested compared to public health data. Where two active substances are indicated, the first reflects the results of studies on duck and goose samples, and the second reflects the results of studies on the active substance in human health.

We were able to compare our results with human resistance data in the Dél-Alföld region (Figure 8). For certain groups of active substances, we had data for different active substances or groups in human health than in animal health, so in these cases, we matched them. Resistance to amoxicillin was much lower in ducks (19.2%) and geese (26.3%) than that of ampicillin in humans (45.4%). For cephalosporins, the resistance rate in humans was just 2.8%. It was similarly low for ceftriaxone in ducks (5.8%), and 15.8% resistant strains were identified in geese. The duck samples showed 1.9% resistance, and the geese samples showed 5.3% resistance to imipenem. The 28.8% resistance to doxycycline in ducks was very similar to that seen with tetracyclines in geese (26.3%) and in public health (36.9).

However, resistance to chloramphenicol in public health was much higher (15.6%) than the resistance in ducks (1.9%) and geese (0.0%). Among fluoroquinolones, ciprofloxacin showed 0.0% resistance in ducks and geese, compared to the public health drug pefloxacin, which showed 22.3% resistance. Resistance to potentiated sulfonamides was much higher in animal health, with 57.7% in ducks and 52.6% in geese, compared to 6.5% in public health.

## 4. Discussion

Multi-resistant strains of *Salmonella enterica* were described in the 1990s and 2000s and have since become prevalent in both human and animal health worldwide, including antibiotic-resistant strains such as fluoroquinolones and third-generation cephalosporins [38,39,40]. Although there has been a significant *Salmonella* eradication program in the poultry sector in Europe since, it did not cover waterfowl, which still pose a significant public health risk. However, at an international level, the widespread use of antimicrobials has increased dramatically [41]. In the European Union alone, the use of tetracycline on livestock farms accounted for 37.0% of all antimicrobials used, and in Canada, 1.5 million kg of antimicrobials were used in intensive livestock production in 2014 [42]. This overuse of antibiotics plays a key role in the development of antibiotic resistance in pathogens, leading to serious economic losses [43]. In this experiment, antibiotic susceptibility testing of a total of 71 *Salmonella* strains isolated from clinical cases was performed between 2022–2023. Although the majority of the examined strains were sensitive to the tested antimicrobial agents, and the significant increase in resistance observed in *Salmonella* strains is not typically compared to that seen in *Escherichia coli*, the decrease in sensitivity to certain critical agents is certainly concerning. This suggests that the sector requires much stricter regulations regarding antibiotic use from the authorities. The sector’s overall antibiotic use, the resulting environmental burden, and the interactions between these factors—farm-to-fork and ultimately to hospital-acquired nosocomial infections—highlight numerous critical points that emphasize the need to preserve the effectiveness of antimicrobial agents for future generations.

In the case of *Salmonella* spp. isolated from waterfowl, there is limited comparative literature available on the effectiveness of ceftiofur, azithromycin, and minocycline, highlighting the need for further research. For ceftriaxone, our study observed resistance rates of 5.8% for ducks and 15.8% for geese. Other studies reported ceftriaxone resistance rates of 71.5% for ducks and 4.3% for geese [44] and 7.1% for ducks and 4.3% for geese [45]. This variation suggests that resistance to ceftriaxone may be influenced by regional factors or differences in sample populations. However, the resistance observed against this class of agents can also be attributed to cross-resistance arising from the use of other authorized agents in poultry. Similarly, we found a 15.8% resistance rate to cefotaxime in geese, which aligns closely with the findings of Cao et al. [46], and 7.9% overall in both types of bird [47]. Similarly, we found resistance to cefotaxime at 15.8% in geese, which is comparable to Cao et al.’s findings for cefotaxime resistance in geese [46]. This consistency underscores the potential stability of cefotaxime resistance in waterfowl, yet it still necessitates ongoing surveillance. Cephalosporins are critically important agents in the hospital treatment of human *Salmonella* infections, particularly in cases of potential septicemia; however, the level of resistance observed against them is concerning. This fact is especially important given that the use of cephalosporins in poultry is not authorized. Therefore, the high level of resistance against these agents may result either from their misuse or from cross-resistance arising from the use of other antimicrobial agents. The similar structure, identical mode of action, and mechanism of effect of the two antibiotic classes may contribute to the development of cross-resistance. Additionally, genes inherited within the same gene cassette could result in combined resistance to multiple antibiotic classes. A deeper understanding of these mechanisms necessitates further in vitro investigations, such as using the MEGA-plate method [48].

For ciprofloxacin, we found no resistant strains in either ducks or geese. Whilst this is similar to Cao et al.’s report of 4.8% resistance in geese [49], it contrasts with Eid et al.’s report of 100% resistance to enrofloxacin [44], Guan et al.’s report of 81.0% resistance in waterfowl [47], and Niu et al.’s description of enrofloxacin resistance of 33.7% [50]. Fluoroquinolones are critically important antibiotics that we reserve for human inpatient care; therefore, their use should be significantly reduced. The fact that we did not observe resistance to ciprofloxacin in our study results is significant because some of the enrofloxacin used in veterinary medicine is metabolized to ciprofloxacin in the animal’s body, which may contribute to the development of resistance against it. Nonetheless, the extent and significance of fluoroquinolone use in the poultry sector are undeniable, and their usage must be significantly reduced to preserve their effectiveness for human inpatient care. The active metabolite ciprofloxacin, formed during the metabolism of enrofloxacin widely used in poultry, has unquestionable human relevance due to its role in the development of resistance [51].

For potentiated sulfonamide, our results showed 57.7% resistance in ducks and 52.6% in geese. However, Guan et al. reported an overall resistance of just 7.1% [47]. Nevertheless, Kim et al. reported 39.7% resistance in ducks [52], and Cao et al. and Eid et al. reported even higher resistance to potentiated sulfonamide, at 81.0% [46] and 86.0% [44] in geese, respectively. These discrepancies could indicate differences in the usage patterns of these drugs or possible variations in bacterial strain resistance mechanisms.

In our study, resistance to imipenem was observed at 1.9% in ducks and 5.3% in geese. This is compared with Guan et al.’s finding of 12.0% imipenem resistance in waterfowl [47]. This suggests that imipenem remains largely effective against *Salmonella*, but its use should be limited to human healthcare. Although imipenem is an antibiotic reserved for human health care, with very low resistance levels observed, these values should be interpreted with caution. The well-known issue of its stability in aqueous solutions makes it difficult to assess accurately.

Resistance to chloramphenicol was detected as 1.9% in ducks, with all strains of goose origin being susceptible, indicating limited resistance development against this antibiotic. In contrast, florfenicol resistance shows more variability, Guan et al. reported 3.2% resistance [47], Cao et al. described 27.3% resistance, Yang et al. found 10.2% resistance in ducks [53], and Cao et al. described 76.2% resistance in geese [46]. For chloramphenicol, it is important to highlight that although it is a broad-spectrum agent and has retained its efficacy, its use in food-producing animals is prohibited. This is because it can cause immediate aplastic anemia in sensitive individuals, with even a single molecule being sufficient to trigger this reaction. Furthermore, no maximum residue level can be established to determine when it is fully eliminated from the body. It is possible that its efficacy is related to the fact that it is not used at all in the sector, similar to florfenicol, which is likely not used frequently due to its exceptionally high effectiveness.

Finally, for amoxicillin–clavulanic acid, 19.2% resistance was found in ducks and 26.3% in geese, compared to 1.5% described by Kim et al. [52]. Similarly, doxycycline resistance in our study was 28.8% resistance in ducks and 26.3% in geese, with Cao et al. reporting 46.8% resistance [49], and Vo et al. describing 5.8% resistance [54]. Levofloxacin resistance in our samples was relatively low, at 9.6% in ducks and 5.3% in geese, which is lower than the 22.8% reported by Niu et al. [50]. These findings suggest that, while resistance profiles are generally similar between ducks and geese, there are significant variations that could be influenced by factors such as regional antibiotic use practices or the specific bacterial strains present. However, disinfection protocols and proper immunization can significantly influence the extent of antibiotic use and the criteria for selecting active substances. Differences in the resistance gene pool of commensal strains, resulting from varying environmental pressures, may also play a role. Commensal strains are proven to act as natural reservoirs of resistance. In the future, it would be worthwhile to begin mapping commensal strains on livestock farms, especially since members of the Enterobacteriaceae family are particularly prone to horizontal gene transfer of resistance genes, not only within species but also between different species.

It is important to highlight the limitations of comparability. On an international level, very few studies focus specifically on waterfowl, and those that do are predominantly from Asia. Most of these studies investigate ducks, which can be explained by their increasing significance in Asia, while we found only a single standalone study on geese. The timing of sample collection also affects the results; although most studies were conducted within the last 5 years, some included samples collected 15–20 years ago.

The most significant limiting factor lies in the methodology, as most studies still rely on the now outdated disk diffusion method. This approach does not correlate well with the microdilution method we used. Only two studies examined strain susceptibility using microdilution. These findings underscore the critical need for surveillance and monitoring studies in this sector.

A correlation analysis on resistance measures to the active substances showed a strong positive correlation for duck samples in several cases, but a lower correlation for goose samples. This may be explained by the smaller number of samples from geese. A strong positive correlation was observed between AMEG A and AMEG B categories, regarding the rates of resistance to the active substances observed for each AMEG category. The analysis of correlation helps to understand the relationships between bacterial isolation sites and antibiotic resistance patterns and could potentially be useful for clinical decision-making. These strong positive correlations suggest that these antibiotics exhibit similar resistance patterns, and resistance to one antibiotic is highly likely to predict resistance to another. It is essential to mention the sample size as a limitation of the study, particularly since it was lower in the case of geese. In a correlational study, we obtain a more accurate picture with a larger sample size. Therefore, in the future, we recognize the need and plan to continuously collect isolated strains, expanding this to include samples received from multiple reference laboratories. Additionally, we aim to examine annual cycles and compare the results to identify temporal trends, supplemented by human resistance data. The main limitations of the correlation analyses are the sample size and the restricted generalizability of the results, as certain agents are not used at all in the poultry sector. Our findings are more indicative in nature, representing the study period and geographical coverage, and should not be interpreted as causal relationships. In the future, it would be valuable to complement these analyses with genetic studies.

Comparing our results with the susceptibility of strains isolated from human cases, we found that the resistance rate to ampicillin was much higher in public health (45.4%) among penicillins (19.2% in ducks and 26.3% in geese). Among Brazilians, a rate of 23.6% resistance has been reported [55], and an even higher resistance rate of 57.7% has been described in China [56]. However, for ceftriaxone, the resistance rate was higher in geese samples (15.8%) and in ducks’ samples (5.8%) than in human samples (2.8%). Talukder et al. detected a resistance of 1.1% in South Asia in human samples [57]. For quinolones and fluoroquinolones, our experiment found much lower levels of resistance in waterfowl than in humans. No resistant strain was identified for ciprofloxacin in our study. However, human samples showed 22.3% resistance to pefloxacin, and Talukder et al. reported 7.6% resistance in humans [57]. Similarly, resistance to chloramphenicol was observed at a low rate only in ducks (1.9%), whereas our study showed that 15.6% of human samples were resistant, and Talukder et al. reported 22.5% resistance [57]. For potentiated sulfonamide, both ducks (57.7%) and geese (52.6%) showed high levels of resistance compared to humans (6.5%), although Talukder et al. found 32.9% resistance in South Asia [57].

Comparing human data with veterinary data in a given area provides a good basis for comparing the resistance profiles of antibiotics used in animal health and active substances used in public health, thus establishing the One Health principle. Samples from clinical cases are associated with higher AMR rates [58,59]. The comparison of regional differences, especially through repeated surveys over time, can help identify trends that may inform the selection of antibiotics in cases of zoonotic human infections. From a legislative perspective, the global rise in multidrug-resistant strains highlights the need to consider making mandatory monitoring programs a standard practice, which would represent a significant step forward. Mapping regional variations is crucial for fine-tuning national antibiotic usage policies. In public health, significant differences in regional antimicrobial resistance levels have been well documented [60]. However, in veterinary health, this issue is more complex due to the unrestricted movement of animal products to consumers across regions. Additionally, the regional clustering of specialized human healthcare services often results in the movement of patients between regions, further complicating the analysis of regional antimicrobial resistance trends.

Limitations of our study include the relatively small sample size and the lack of a wide geographical distribution of samples due to the concentration of the sector. Our results were intended not only to complement the epidemiological data but also to provide practical relevance as a guideline for the application of antimicrobials in waterfowl. Based on the resistance patterns observed, it is crucial to prioritize the use of antimicrobials that show low resistance rates in treating waterfowl infections. These drugs should be considered first-line treatments where applicable. Additionally, regular monitoring of antimicrobial resistance in waterfowl populations should be implemented, particularly for antibiotics like ceftriaxone, potentiated sulfonamides, and doxycycline, where resistance rates are variable and can reach concerning levels. Such monitoring will help in updating treatment protocols and ensuring the continued efficacy of these drugs.

Given the varying resistance rates observed for broad-spectrum antibiotics like florfenicol and amoxicillin–clavulanic acid, it is recommended to limit their use to cases where susceptibility testing confirms their efficacy, helping to prevent further resistance development. Implementing antibiotic stewardship programs in regions with high resistance rates is essential to reduce the unnecessary use of antimicrobials and to promote alternative strategies, such as vaccination and improved biosecurity measures, to control infections in waterfowl populations. It has already been proven that the periodic rotation of antibiotics leads to a decline in resistant strains, as pathogens, striving to minimize energy expenditure, tend to discard unnecessary resistance genes. As a result, the so-called wild-type strains begin to dominate the population once again. Understanding these mechanisms plays a key role in reducing resistance, with proper education serving as the cornerstone of this effort.

Finally, additional studies are needed to explore the effectiveness of less commonly used antibiotics, such as ceftiofur, azithromycin, and minocycline, for which limited comparative data is available. Understanding their potential role in waterfowl treatment could expand the options for managing resistant infections and contribute to more effective antimicrobial strategies in this sector. *Salmonella* continues to pose a significant human infection risk in the waterfowl sector; therefore, new European Union directives are needed to effectively reduce *Salmonella* carriage in this sector. The waterfowl sector is a neglected area in this regard, despite its undeniable economic significance. Therefore, the mandatory implementation and enforcement of Salmonella control and reduction programs, similar to those in place for chicken and turkey flocks, would be a timely and necessary step for the waterfowl industry as well.

## 5. Conclusions

Overall, the waterfowl sector in Hungary is concentrated in the Dél-Alföld region and the patterns of AMR in both ducks and geese are very similar. The high resistance to potentiated sulfonamides in this region is likely due to their overuse over several decades.

Our results reflect the animal health status of AMR and the spread of resistance. However, one possible explanation could be the significantly larger sample size in human cases, which is an order of magnitude greater. Therefore, in order to ascertain the true extent of AMR in waterfowl, and its effects on public health, it is essential to carry out similar surveys with a larger number of representative samples, complemented by periodic repeat testing. In addition, to investigate the underlying causes, it may be worthwhile complementing the surveys with next-generation sequencing for strains showing multidrug resistance. Our results suggest that it would be worthwhile to expand our investigations in the future by also targeting the reduction of commensal strains, as these strains may serve as natural reservoirs of resistance.

## Figures and Tables

**Table 1:** Frequency table of the minimum inhibitory concentration (MIC) values of the active substances obtained from duck samples (*n* = 52) with breakpoints. The top row for each active substance represents the number of items, and the bottom row shows the percentage for each. The vertical red line marks the breakpoint of resistance. The grey parts did not occur.

Antibiotic	^1^ BP *	0.007	0.015	0.03	0.06	0.125	0.25	0.5	1	2	4	8	16	32	64	128	256	512	1024	MIC_50_	MIC_90_	^2^ ECOFF
	µg/mL																			µg/mL		
Azithromycin	32										7	43	1	0	0	0	0	1		8	8	16
											13.5%	82.7%	1.9%	0.0%	0.0%	0.0%	0.0%	1.9%				
Cefotaxime	4	1	0	3	11	5	7	8	3	0	0	7	6	0	1					0.25	16	4
		1.9%	0.0%	5.8%	21.2%	9.6%	13.5%	15.4%	5.8%	0.0%	0.0%	13.5%	11.5%	0.0%	1.9%							
Ceftiofur	2	1	0	0	0	0	1	18	18	0	6	6	2							1	8	2
		1.9%	0.0%	0.0%	0.0%	0.0%	1.9%	34.6%	34.6%	0.0%	11.5%	11.5%	3.8%									
Ceftriaxone	1		1	6	17	9	5	3	5	2	0	0	1	2	0	0	0	1		0.125	2	0.25
			1.9%	11.5%	32.7%	17.3%	9.6%	5.8%	9.6%	3.8%	0.0%	0.0%	1.9%	3.8%	0.0%	0.0%	0.0%	1.9%				
Ciprofloxacin	0.06	20	10	4	8	4	2	3	1											0.015	0.25	0.125
		38.5%	19.2%	7.7%	15.4%	7.7%	3.8%	5.8%	1.9%													
Doxycycline	4									10	19	7	3	9	4					4	32	8
										19.2%	36.5%	13.5%	5.8%	17.3%	7.7%							
Imipenem	1					6	8	13	21	1	0	1	0	2						0.5	1	1
						11.5%	15.4%	25.0%	40.4%	1.9%	0.0%	1.9%	0.0%	3.8%								
Chloramphenicol	8										43	8	0	1						4	8	16
											82.7%	15.4%	0.0%	1.9%								
Levofloxacin	0.125	2	4	14	16	1	1	5	3	1	1	2	2							0.06	2	0.25
		3.8%	7.7%	26.9%	30.8%	1.9%	1.9%	9.6%	5.8%	1.9%	1.9%	3.8%	3.8%									
^3^ Potentiated sulfonamide	4/76							5	2	14	8	4	5	2	3	3	4	1	1	4	256	1
								9.6%	3.8%	26.9%	15.4%	7.7%	9.6%	3.8%	5.8%	5.8%	7.7%	1.9%	1.9%			

* BP—breakpoint; ^1^ CLSI; ^2^ EUCAST; ^3^ trimpehrprime–sulpphamethoxazole 1:19 ratio.

**Table 2:** Frequency table of the minimum inhibitory concentration (MIC) values of the active substances obtained in the breakpoint test of goose samples (*n* = 19). The top row for each active substance represents the number of items, and the bottom row shows the percentage for each. The vertical red line marks the breakpoint of resistance. The grey parts did not occur.

Antibiotic	^1^ BP *	0.007	0.015	0.03	0.06	0.125	0.25	0.5	1	2	4	8	16	32	64	128	256	512	1024	MIC_50_	MIC_90_	^2^ ECOFF
	µg/mL																			µg/mL		
Azithromycin	32								1	0	4	13	1							8	8	16
									5.3%	0.0%	21.1%	68.4%	5.3%									
Cefotaxime	4			2	5	1	1	3	4	0	1	2								0.5	4	4
				10.5%	26.3%	5.3%	5.3%	15.8%	21.1%	0.0%	5.3%	10.5%										
Ceftiofur	8	3	0	1	0	1	3	5	2	0	1	2	1							0.5	8	2
		15.8%	0.0%	5.3%	0.0%	5.3%	15.8%	26.3%	10.5%	0.0%	5.3%	10.5%	5.3%									
Ceftriaxone	4		1	2	4	4	1	1	1	3	0	0	0	2						0.125	2	0.25
			5.3%	10.5%	21.1%	21.1%	5.3%	5.3%	5.3%	15.8%	0.0%	0.0%	0.0%	10.5%								
Ciprofloxacin	1	11	4	1	1	1	1													0.007	0.06	0.125
		57.9%	21.1%	5.3%	5.3%	5.3%	5.3%															
Doxycycline	16						1	0	2	3	7	1	3	1	1					4	16	8
							5.3%	0.0%	10.5%	15.8%	36.8%	5.3%	15.8%	5.3%	5.3%							
Imipenem	4					1	2	11	1	2	0	0	0	1						0.5	2	1
						5.3%	10.5%	57.9%	5.3%	10.5%	0.0%	0.0%	0.0%	5.3%								
Chloramphenicol	32								2	0	13	4								4	8	16
									10.5%	0.0%	68.4%	21.1%										
Levofloxacin	2	3	4	6	3	1	0	1	0	0	0	0	1							0.03	0.125	0.25
		15.8%	21.1%	31.6%	15.8%	5.3%	0.0%	5.3%	0.0%	0.0%	0.0%	0.0%	5.3%									
^3^ Potentiated sulfonamide	4/76							4	0	5	3	4	0	0	0	1	0	0	2	4	128	1
								21.1%	0.0%	26.3%	15.8%	21.1%	0.0%	0.0%	0.0%	5.3%	0.0%	0.0%	10.5%			

* BP—breakpoint; ^1^ CLSI; ^2^ EUCAST; ^3^ trimpehrprime–sulpphamethoxazole 1:19 ratio.

## Data Availability

The original contributions presented in this study are included in the article/supplementary material. Further inquiries can be directed to the corresponding author.

## Supplementary Materials

The following supporting information can be downloaded at https://www.mdpi.com/article/10.3390/microorganisms12122462/s1. Table S1: The determination of the minimum inhibitory concentration (MIC) values of antibiotic agents important from an animal and public health perspective was carried out for *Salmonella* strains isolated from ducks. This included the organ origin of each sample, and the geographic origin by town and region; Table S2: The determination of the minimum inhibitory concentration (MIC) values of antibiotic agents important from an animal and public health perspective was carried out for *Salmonella* strains isolated from ducks. This included the organ origin of each sample, and the geographic origin by town and region (continued); Table S3: The determination of the minimum inhibitory concentration (MIC) values of antibiotic agents important from an animal and public health perspective was carried out for *Salmonella* strains isolated from geese. This included the organ origin of each sample, and the geographic origin by town and region; Table S4: The determination of the minimum inhibitory concentration (MIC) values of antibiotic agents important from an animal and public health perspective was carried out for *Salmonella* strains isolated from geese. This included the organ origin of each sample, and the geographic origin by town and region (continued); Table S5: Statistical analysis of the resistance profiles of samples derived from ducks and geese, comparing the resistance levels of the two animal species for each antibiotic agent.
