# Supplementary material for: Antimicrobial Susceptibility Profiles of Salmonella spp. Isolates from Clinical Cases of Waterfowl in Hungary Between 2022 and 2023
Source: Microorganisms. 2024 Nov 29;12(12):2462. doi: 10.3390/microorganisms12122462 (PMC11676221; doi:10.3390/microorganisms12122462)
Supplement: Supplementary file 1 [file microorganisms-12-02462-s001.zip › Supplementary Materials.pdf]

Table S1. The determination of the minimum inhibitory concentration (MIC) values of antibiotic agents (with breakpoints) important from an animal and public health perspective was carried out for Salmonella strains isolated from ducks. This included the organ origin of each sample, and the geographic origin by town and region.

MALDI-TOF	Log (score)	Isolation	AMC	CTO	AZI	CHP	CTM	LEV	CIP	CTR	IMI	MIN	DOX	PSA	Town	Region
Salmonella sp. (Enteritidis)	2.37	bone marrow	0.5	0.5	8	4	0.06	0.03	0.015	0.03	1	2	64	4	Rozsály	Észak-Alföld
Salmonella sp. (Typhimurium)	2.33	bone marrow	2	4	8	4	8	0.03	0.015	0.015	1	4	8	4	Csikéria	Dél-Alföld
Salmonella sp. (Typhimurium)	2.48	bone marrow	64	16	8	8	8	0.125	0.015	1	1	4	4	512	Kiskunmajsa	Dél-Alföld
Salmonella sp. (Enteritidis)	2.45	bone marrow	512	1	8	4	0.125	0.06	0.007	16	1	2	32	32	Tázlár	Dél-Alföld
Salmonella sp. (Dublin)	2.32	bone marrow	1	0.007	4	4	0.06	0.5	0.125	0.125	1	8	16	16	Tiszakécske	Dél-Alföld
Salmonella sp. (Typhimurium)	2.48	bone marrow	8	0.5	8	4	1	0.03	0.06	0.25	0.125	4	32	16	Bócsa	Dél-Alföld
Salmonella sp. (Typhimurium)	2.35	bone marrow	8	16	8	4	41	0.06	0.015	1	1	2	4	128	Soltvadkert	Dél-Alföld
Salmonella sp. (Enteritidis)	2.43	bone marrow	0.5	0.5	8	4	0.25	16	0.007	0.03	0.125	4	2	16	Balotaszállás	Dél-Alföld
Salmonella sp. (Enteritidis)	2.41	bone marrow	2	1	8	8	0.25	0.5	0.5	0.125	1	4	4	2	Csólyospálos	Dél-Alföld
Salmonella sp. (Enteritidis)	2.2	bone marrow	2	0.5	8	4	0.25	1	0.015	32	1	4	2	1	Csólyospálos	Dél-Alföld
Salmonella sp. (Enteritidis)	2.32	bone marrow	2	8	8	4	8	0.06	0.06	0.06	8	4	2	2	Kiskunmajsa	Dél-Alföld
Salmonella sp. (Anatum)	2.39	liver	0.5	1	8	4	0.06	0.06	0.007	0.06	0.5	4	2	0.5	Csikéria	Dél-Alföld
Salmonella sp. (Enteritidis)	2.3	bone marrow	4	1	8	4	0.5	0.03	0.125	0.125	1	2	4	4	Szank	Dél-Alföld
Salmonella sp. (Enteritidis)	2.5	bone marrow	4	0.5	8	4	0.06	0.25	1	0.125	0.5	4	4	8	Forráskút	Dél-Alföld
Salmonella sp. (Enteritidis)	2.33	bone marrow	16	1	8	4	0.5	0.06	0.007	0.06	0.25	4	32	8	Soltvadkert	Dél-Alföld
Salmonella sp. (Typhimurium)	2.38	bone marrow	8	1	8	4	0.03	0.015	0.007	0.5	0.5	2	8	128	Soltvadkert	Dél-Alföld
Salmonella sp. (Typhimurium)	2.42	liver	2	1	4	4	0.25	8	0.007	0.125	1	4	8	2	Forráskút	Dél-Alföld
Salmonella sp. (Enteritidis)	2.49	pericardium	64	0.5	16	4	0.06	0.03	0.007	32	1	4	64	256	Szank	Dél-Alföld
Salmonella sp. (Anatum)	2.33	liver	64	8	8	4	8	0.015	0.06	0.06	1	4	32	256	Csólyospálos	Dél-Alföld
Salmonella sp. (Typhimurium)	2.3	bone marrow	4	8	8	4	8	0.03	0.03	0.06	1	2	4	2	Kiskunmajsa	Dél-Alföld
Salmonella sp. (Typhimurium)	2.43	bone marrow	4	0.5	8	4	0.5	0.03	0.007	0.06	1	2	4	2	Nyárlőrinc	Dél-Alföld
Salmonella sp. (Hadar)	2.36	bone marrow	4	0.5	4	4	0.007	4	0.25	0.125	0.125	4	32	4	Bordány	Dél-Alföld
Salmonella sp. (Enteritidis)	2.44	bone marrow	4	8	8	4	16	0.06	0.015	0.125	32	4	4	2	Kiskunmajsa	Dél-Alföld
Salmonella sp. (Enteritidis)	2.55	bone marrow	4	0.5	4	4	0.5	16	0.007	0.06	32	2	4	2	Kiskunhalas	Dél-Alföld
Salmonella sp. (Anatum)	2.41	bone marrow	2	0.5	8	4	0.03	0.03	0.03	0.03	0.25	2	4	4	Csikéria	Dél-Alföld
Salmonella sp. (Typhimurium)	2.3	bone marrow	16	0.5	4	4	0.125	8	0.007	0.5	0.125	2	8	256	Kiskunfélegyháza	Dél-Alföld

Salmonella sp. (Typhimurium)	2.36	bone marrow	2	8	8	4	8	0.06	0.06	2	0.25	2	4	8	Zsámbok	Közép-Magyarország
Salmonella sp. (Typhimurium)	2.38	bone marrow	4	1	8	4	0.125	0.06	0.007	1	1	4	4	2	Csikéria	Dél-Alföld
Salmonella sp. (Typhimurium)	2.42	bone marrow	4	1	8	4	0.5	0.06	0.015	0.125	0.25	4	4	4	Csólyospálos	Dél-Alföld
Salmonella sp. (Typhimurium)	2.3	bone marrow	2	1	8	4	0.06	0.03	0.007	0.06	0.25	4	8	8	Szank	Dél-Alföld
Salmonella sp. (Dublin)	2.34	liver	0.5	4	8	4	16	0.06	0.06	0.06	0.25	4	2	1	Kiskunfélegyháza	Dél-Alföld
Salmonella sp. (Enteritidis)	2.42	bone marrow	4	1	8	8	0.25	0.03	0.03	1	0.25	2	64	64	Kiskunmajsa	Dél-Alföld
Salmonella sp. (Enteritidis)	2.48	bone marrow	0.5	0.5	512	32	0.06	0.06	0.125	512	1	64	32	2	Zsombó	Dél-Alföld
Salmonella sp. (Typhimurium)	2.44	liver	16	4	8	4	16	0.06	0.007	0.03	0.125	4	64	16	Szank	Dél-Alföld
Salmonella sp. (Typhimurium)	2.45	brain chamber	8	1	8	4	0.5	0.03	0.007	0.25	0.5	1	8	16	Zsombó	Dél-Alföld
Salmonella sp. (Enteritidis)	2.4	bone marrow	32	0.5	8	4	0.06	0.015	0.015	0.06	1	2	16	32	Szank	Dél-Alföld
Salmonella sp. (Anatum)	2.42	liver	2	4	8	4	16	0.06	0.007	0.03	0.5	4	2	0.5	Kunfehértó	Dél-Alföld
Salmonella sp. (Enteritidis)	2.39	liver	0.5	4	8	4	16	0.03	0.06	0.06	0.5	4	2	2	Kiskunfélegyháza	Dél-Alföld
Salmonella sp. (Dublin)	2.3	bone marrow	32	1	8	8	0.25	1	0.5	1	1	4	32	64	Kiskunmajsa	Dél-Alföld
Salmonella sp. (Enteritidis)	2.32	bone marrow	32	1	8	8	0.25	0.03	0.03	0.5	1	2	16	64	Csólyospálos	Dél-Alföld
Salmonella sp. (Typhimurium)	2.45	bone marrow	8	1	4	4	0.5	1	0.015	0.06	0.125	4	32	1024	Kiskunmajsa	Dél-Alföld
Salmonella sp. (Choleraesuis)	2.36	bone marrow	2	1	4	4	0.5	2	0.015	0.125	1	2	4	0.5	Csólyospálos	Dél-Alföld
Salmonella sp. (Typhimurium)	2.38	bone marrow	32	0.5	8	4	0.06	0.06	0.06	2	1	2	32	256	Kelebia	Dél-Alföld
Salmonella sp. (Anatum)	2.37	brain chamber	2	0.5	8	4	0.06	0.5	0.125	0.25	2	8	4	128	Csólyospálos	Dél-Alföld
Salmonella sp. (Typhimurium)	2.2	bone marrow	2	0.5	8	4	0.06	0.03	0.007	0.06	0.5	4	4	4	Soltvadkert	Dél-Alföld
Salmonella sp. (Dublin)	2.42	bone marrow	16	1	8	4	0.125	0.5	0.5	0.06	0.5	4	2	0.5	Kiskunmajsa	Dél-Alföld
Salmonella sp. (Enteritidis)	2.47	bone marrow	0.5	1	8	8	0.125	0.06	0.007	0.06	0.5	2	2	0.5	Szank	Dél-Alföld
Salmonella sp. (Enteritidis)	2.37	bone marrow	2	0.5	8	8	1	0.007	0.007	0.025	0.5	4	4	2	Kiskunmajsa	Dél-Alföld
Salmonella sp. (Enteritidis)	2.37	bone marrow	2	0.5	8	8	1	0.007	0.007	0.06	0.5	2	4	4	Tázlár	Dél-Alföld
Salmonella sp. (Enteritidis)	2.4	liver	2	0.25	8	4	0.03	0.5	0.25	0.25	0.5	8	8	2	Csólyospálos	Dél-Alföld
Salmonella sp. (Dublin)	2.51	liver	2	8	8	4	8	0.015	0.06	0.25	0.25	2	4	2	Csólyospálos	Dél-Alföld
Salmonella sp. (Anatum)	2.46	liver	0.5	4	8	4	16	0.06	0.007	0.06	0.5	2	2	2	Kiskunfélegyháza	Dél-Alföld
**MIC_{50}**			**1**	**8**	**4**	**4**	**0.25**	**0.06**	**0.015**	**0.125**	**0.5**	**4**	**4**	**4**		
**MIC_{90}**			**8**	**8**	**32**	**8**	**16**	**2**	**0.25**	**2**	**1**	**4**	**32**	**256**		

CT – ceftiofur; AZI – azithromycin; CHP – chloramphenicol; CTM – cefotaxime; LEV – levofloxacin; CIP – ciprofloxacin; CTR – ceftriaxone; IMI – imipenem; DOX – doxycycline; PSA – potentiated sulfonamide (trimethoprim – sulfamethoxazole 1:19 ratio)

Table S2. The determination of the minimum inhibitory concentration (MIC) values of antibiotic agents (without breakpoints) important from an animal and public health perspective was carried out for Salmonella strains isolated from ducks. This included the organ origin of each sample, and the geographic origin by town and region (continued).

MALDI-TOF	Log (score)	Isolation	AMX	NEO	SPE	FLO	TIL	TIA	LIN	ENR	COL	Town	Region
Salmonella sp. (Enteritidis)	2.37	bone marrow	4	32	128	4	1024	1024	1024	0.03	1024	Rozsály	Észak-Alföld
Salmonella sp. (Typhimurium)	2.33	bone marrow	0.5	64	256	8	1024	1024	1024	0.015	8	Csikéria	Dél-Alföld
Salmonella sp. (Typhimurium)	2.48	bone marrow	512	16	256	4	1024	512	1024	1	8	Kiskunmajsa	Dél-Alföld
Salmonella sp. (Enteritidis)	2.45	bone marrow	512	512	512	256	512	256	1024	0.06	2	Tázlár	Dél-Alföld
Salmonella sp. (Dublin)	2.32	bone marrow	2	8	128	4	512	256	512	1	0.5	Tiszakécske	Dél-Alföld
Salmonella sp. (Typhimurium)	2.48	bone marrow	1024	512	128	16	1024	1024	1024	0.015	0.015	Bócsa	Dél-Alföld
Salmonella sp. (Typhimurium)	2.35	bone marrow	32	64	128	8	512	256	1024	0.03	1	Soltvadkert	Dél-Alföld
Salmonella sp. (Enteritidis)	2.43	bone marrow	16	32	256	8	1024	1024	1024	0.125	0.003	Balotaszállás	Dél-Alföld
Salmonella sp. (Enteritidis)	2.41	bone marrow	16	16	256	16	1024	512	1024	0.5	16	Csólyospálos	Dél-Alföld
Salmonella sp. (Enteritidis)	2.2	bone marrow	8	64	256	16	512	512	1024	0.5	32	Csólyospálos	Dél-Alföld
Salmonella sp. (Enteritidis)	2.32	bone marrow	8	4	128	16	512	128	512	0.03	1	Kiskunmajsa	Dél-Alföld
Salmonella sp. (Anatum)	2.39	liver	0.5	8	64	2	1024	256	1024	0.03	0.25	Csikéria	Dél-Alföld
Salmonella sp. (Enteritidis)	2.3	bone marrow	2	16	128	8	1024	128	512	0.03	4	Szank	Dél-Alföld
Salmonella sp. (Enteritidis)	2.5	bone marrow	2	16	128	4	512	128	1024	0.03	1	Forráskút	Dél-Alföld
Salmonella sp. (Enteritidis)	2.33	bone marrow	4	4	32	128	512	256	1024	0.03	1	Soltvadkert	Dél-Alföld
Salmonella sp. (Typhimurium)	2.38	bone marrow	32	16	256	8	256	256	512	16	8	Soltvadkert	Dél-Alföld
Salmonella sp. (Typhimurium)	2.42	liver	2	32	128	8	1024	256	1024	0.06	8	Forráskút	Dél-Alföld
Salmonella sp. (Enteritidis)	2.49	pericardium	64	64	128	128	512	256	512	16	32	Szank	Dél-Alföld
Salmonella sp. (Anatum)	2.33	liver	64	32	128	128	512	256	1024	16	16	Csólyospálos	Dél-Alföld
Salmonella sp. (Typhimurium)	2.3	bone marrow	1	16	128	4	1024	256	1024	0.03	8	Kiskunmajsa	Dél-Alföld
Salmonella sp. (Typhimurium)	2.43	bone marrow	2	32	128	8	1024	256	1024	0.03	1	Nyárlőrinc	Dél-Alföld
Salmonella sp. (Hadar)	2.36	bone marrow	2	32	128	4	512	256	512	1	1	Bordány	Dél-Alföld
Salmonella sp. (Enteritidis)	2.44	bone marrow	1	16	128	8	256	256	1024	0.03	2	Kiskunmajsa	Dél-Alföld
Salmonella sp. (Enteritidis)	2.55	bone marrow	1	16	256	8	256	256	1024	0.03	8	Kiskunhalas	Dél-Alföld
Salmonella sp. (Anatum)	2.41	bone marrow	1	4	128	16	512	256	512	0.03	0.125	Csikéria	Dél-Alföld
Salmonella sp. (Typhimurium)	2.3	bone marrow	16	64	512	16	1024	1024	1024	0.015	0.015	Kiskunfélegyháza	Dél-Alföld

Salmonella sp. (Typhimurium)	2.36	bone marrow	2	32	256	8	512	256	1024	0.03	4	Zsámbok	Közép-Magyarország
Salmonella sp. (Typhimurium)	2.38	bone marrow	2	8	256	4	1024	256	512	0.03	8	Csikéria	Dél-Alföld
Salmonella sp. (Typhimurium)	2.42	bone marrow	2	16	128	4	512	512	512	0.06	1	Csólyospálos	Dél-Alföld
Salmonella sp. (Typhimurium)	2.3	bone marrow	2	32	128	4	512	512	512	0.03	8	Szank	Dél-Alföld
Salmonella sp. (Dublin)	2.34	liver	4	16	64	4	1024	256	1024	0.015	16	Kiskunfélegyháza	Dél-Alföld
Salmonella sp. (Enteritidis)	2.42	bone marrow	8	32	512	16	1024	1024	1024	8	0.015	Kiskunmajsa	Dél-Alföld
Salmonella sp. (Enteritidis)	2.48	bone marrow	4	512	128	4	1024	1024	1024	0.25	1024	Zsombó	Dél-Alföld
Salmonella sp. (Typhimurium)	2.44	liver	8	64	64	32	512	1024	1024	16	0.015	Szank	Dél-Alföld
Salmonella sp. (Typhimurium)	2.45	brain chamber	2	64	64	16	512	1024	1024	0.03	2	Zsombó	Dél-Alföld
Salmonella sp. (Enteritidis)	2.4	bone marrow	8	64	64	64	256	1024	1024	0.06	8	Szank	Dél-Alföld
Salmonella sp. (Anatum)	2.42	liver	2	16	32	4	1024	256	512	0.007	8	Kunfehértó	Dél-Alföld
Salmonella sp. (Enteritidis)	2.39	liver	4	16	64	4	1024	256	1024	0.007	4	Kiskunfélegyháza	Dél-Alföld
Salmonella sp. (Dublin)	2.3	bone marrow	4	64	64	16	256	128	1024	8	32	Kiskunmajsa	Dél-Alföld
Salmonella sp. (Enteritidis)	2.32	bone marrow	4	64	128	16	256	256	512	0.25	8	Csólyospálos	Dél-Alföld
Salmonella sp. (Typhimurium)	2.45	bone marrow	1024	8	1024	512	1024	1024	1024	512	0.5	Kiskunmajsa	Dél-Alföld
Salmonella sp. (Choleraesuis)	2.36	bone marrow	4	32	32	4	1024	256	1024	0.25	8	Csólyospálos	Dél-Alföld
Salmonella sp. (Typhimurium)	2.38	bone marrow	64	64	128	32	256	256	512	1	32	Kelebia	Dél-Alföld
Salmonella sp. (Anatum)	2.37	brain chamber	2	64	128	4	256	256	512	0.125	8	Csólyospálos	Dél-Alföld
Salmonella sp. (Typhimurium)	2.2	bone marrow	0.5	32	256	4	1024	512	1024	0.015	8	Soltvadkert	Dél-Alföld
Salmonella sp. (Dublin)	2.42	bone marrow	1024	64	128	4	512	256	1024	0.25	0.06	Kiskunmajsa	Dél-Alföld
Salmonella sp. (Enteritidis)	2.47	bone marrow	1	32	128	8	1024	256	1024	0.03	0.125	Szank	Dél-Alföld
Salmonella sp. (Enteritidis)	2.37	bone marrow	2	32	256	4	1024	256	1024	0.03	0.125	Kiskunmajsa	Dél-Alföld
Salmonella sp. (Enteritidis)	2.37	bone marrow	4	32	256	4	1024	256	1024	0.25	0.5	Tázlár	Dél-Alföld
Salmonella sp. (Enteritidis)	2.4	liver	4	32	128	4	1024	256	512	0.25	0.5	Csólyospálos	Dél-Alföld
Salmonella sp. (Dublin)	2.51	liver	4	32	128	4	512	512	512	0.06	0.5	Csólyospálos	Dél-Alföld
Salmonella sp. (Anatum)	2.46	liver	4	16	64	4	1024	256	1024	0.007	4	Kiskunfélegyháza	Dél-Alföld
**MIC_{50}**			**4**	**32**	**128**	**8**	**512**	**256**	**1024**	**0.03**	**4**		
**MIC_{90}**			**64**	**64**	**256**	**64**	**1024**	**1024**	**1024**	**8**	**32**		

AMX – amoxicillin; AMC – amoxicillin-clavulanic acid (2:1 ratio); NEO – neomycin; SPE – spectinomycin; FLO – florfenicol; TIL – tilozin; TIA – tiamulin; LIN – lincomycin; ENR – enrofloxacin; COL – colistin; MIN – minocycline

Table S3. The determination of the minimum inhibitory concentration (MIC) values of antibiotic agents (with breakpoints) important from an animal and public health perspective was carried out for Salmonella strains isolated from geese. This included the organ origin of each sample, and the geographic origin by town and region.

MALDI-TOF	Log (score)	Isolation	AMC	CTO	AZI	CHP	CTM	LEV	CIP	CTR	IMI	MIN	DOX	PSA	Town	Region
Salmonella sp. (Typhimurium)	2.41	bone marrow	2	4	16	8	8	0.06	0.03	0.06	0.5	4	4	2	Kiskunmajsa	Dél-Alföld
Salmonella sp. (Typhimurium)	2.47	bone marrow	16	16	8	8	8	0.125	0.015	2	0.5	4	4	1024	Csólyospálos	Dél-Alföld
Salmonella sp. (Dublin)	2.35	bone marrow	8	0.007	8	4	0.06	0.03	0.007	0.06	0.5	128	64	0.5	Kunmadaras	Észak-Alföld
Salmonella sp. (Anatum)	2.45	bone marrow	4	0.007	4	4	0.25	0.015	0.007	0.25	2	4	2	2	Csólyospálos	Dél-Alföld
Salmonella sp. (Anatum)	2.38	bone marrow	2	0.5	8	4	0.5	16	0.007	32	32	4	16	4	Albertirsa	Közép-Magyarország
Salmonella sp. (Dublin)	2.51	bone marrow	32	0.5	8	4	0.06	0.03	0.06	2	0.5	4	4	8	Kecel	Dél-Alföld
Salmonella sp. (Typhimurium)	2.47	lungs	16	0.5	4	4	0.06	0.5	0.25	0.125	0.5	4	8	1024	Mátészalka	Észak-Alföld
Salmonella sp. (Typhimurium)	2.49	oviduct	4	0.125	1	1	0.03	0.03	0.007	1	0.5	0.007	0.25	4	Szentes	Dél-Alföld
Salmonella sp. (Typhimurium)	2.33	bone marrow	2	0.03	4	4	0.06	0.03	0.007	0.03	0.125	4	1	2	Algyő	Dél-Alföld
Salmonella sp. (Typhimurium)	2.37	bone marrow	1	0.25	8	4	1	0.015	0.007	0.03	0.03	4	4	8	Jászapáti	Észak-Alföld
Salmonella sp. (Typhimurium)	2.36	liver	32	0.25	8	4	1	0.015	0.007	32	0.5	4	4	128	Gyöngyös	Észak-Magyarország
Salmonella sp. (Typhimurium)	2.48	liver	4	8	8	4	0.5	0.06	0.015	0.125	0.25	4	2	8	Szank	Dél-Alföld
Salmonella sp. (Dublin)	2.44	liver	32	8	4	4	0.5	0.06	0.015	2	0.5	2	32	4	Öcsöd	Észak-Alföld
Salmonella sp. (Typhimurium)	2.39	bone marrow	8	0.5	8	4	0.13	0.015	0.125	0.015	0.25	2	1	8	Kiskunmajsa	Dél-Alföld
Salmonella sp. (Typhimurium)	2.38	liver	1	1	8	1	0.06	0.03	0.015	0.5	2	4	4	2	Rém	Dél-Alföld
Salmonella sp. (Typhimurium)	2.53	bone marrow	2	0.5	8	8	1	0.007	0.007	0.125	0.5	2	2	0.5	Kiskunmajsa	Dél-Alföld
Salmonella sp. (Typhimurium)	2.36	bone marrow	2	0.007	8	4	0.03	0.03	0.007	0.06	0.5	0.5	4	2	Kunbaracs	Dél-Alföld
Salmonella sp. (Enteritidis)	2.4	bone marrow	2	1	8	8	4	0.007	0.007	0.06	1	2	16	0.5	Kiskunfélegyháza	Dél-Alföld
Salmonella sp. (Typhimurium)	2.48	bone marrow	2	0.25	8	4	1	0.007	0.007	0.125	0.5	2	16	0.5	Daruszentmiklós	Közép-Dunántúl
**MIC_{50}**			**4**	**0.5**	**8**	**4**	**0.5**	**0.03**	**0.007**	**0.125**	**0.5**	**4**	**4**	**4**		
**MIC_{90}**			**32**	**8**	**8**	**8**	**4**	**0.125**	**0.06**	**2**	**2**	**4**	**16**	**128**		

CT – ceftiofur; AZI – azithromycin; CHP – chloramphenicol; CTM – cefotaxime; LEV – levofloxacin; CIP – ciprofloxacin; CTR – ceftriaxone; IMI – imipenem; DOX – doxycycline; PSA – potentiated sulfonamide (trimethoprim – sulfamethoxazole 1:19 ratio)

Table S4. The determination of the minimum inhibitory concentration (MIC) values of antibiotic agents (without breakpoints) important from an animal and public health perspective was carried out for Salmonella strains isolated from geese. This included the organ origin of each sample, and the geographic origin by town and region (continued).

MALDI-TOF	Log (score)	Isolation	AMX	NEO	SPE	FLO	TIL	TIA	LIN	ENR	COL	Town	Region
Salmonella sp. (Typhimurium)	2.41	bone marrow	4	32	64	4	1024	256	1024	0.015	1	Kiskunmajsa	Dél-Alföld
Salmonella sp. (Typhimurium)	2.47	bone marrow	2	128	64	4	512	256	1024	0.06	8	Csólyospálos	Dél-Alföld
Salmonella sp. (Dublin)	2.35	bone marrow	1024	64	128	8	1024	512	1024	0.06	0.25	Kunmadaras	Észak-Alföld
Salmonella sp. (Anatum)	2.45	bone marrow	1	8	128	8	256	256	1024	0.03	8	Csólyospálos	Dél-Alföld
Salmonella sp. (Anatum)	2.38	bone marrow	1	64	64	8	128	256	512	8	32	Albertirsa	Közép-Magyarország
Salmonella sp. (Dublin)	2.51	bone marrow	4	64	64	8	256	256	1024	0.06	1	Kecel	Dél-Alföld
Salmonella sp. (Typhimurium)	2.47	lungs	512	4	512	8	1024	512	1024	2	0.06	Mátészalka	Észak-Alföld
Salmonella sp. (Typhimurium)	2.49	oviduct	2	16	128	2	64	128	1024	0.03	0.125	Szentes	Dél-Alföld
Salmonella sp. (Typhimurium)	2.33	bone marrow	1	8	32	2	512	128	1024	0.007	0.015	Algyő	Dél-Alföld
Salmonella sp. (Typhimurium)	2.37	bone marrow	64	4	32	4	512	32	1024	0.007	0.015	Jászapáti	Észak-Alföld
Salmonella sp. (Typhimurium)	2.36	liver	32	32	64	128	1024	128	1024	32	8	Gyöngyös	Észak-Magyarország
Salmonella sp. (Typhimurium)	2.48	liver	2	32	64	4	512	128	512	0.007	2	Szank	Dél-Alföld
Salmonella sp. (Dublin)	2.44	liver	64	32	128	8	512	128	512	0.007	4	Öcsöd	Észak-Alföld
Salmonella sp. (Typhimurium)	2.39	bone marrow	256	64	128	512	512	256	1024	0.015	0.5	Kiskunmajsa	Dél-Alföld
Salmonella sp. (Typhimurium)	2.38	liver	2	32	256	4	1024	512	1024	0.03	16	Rém	Dél-Alföld
Salmonella sp. (Typhimurium)	2.53	bone marrow	2	16	125	4	512	512	512	0.03	0.5	Kiskunmajsa	Dél-Alföld
Salmonella sp. (Typhimurium)	2.36	bone marrow	4	64	256	8	512	512	512	0.125	0.5	Kunbaracs	Dél-Alföld
Salmonella sp. (Enteritidis)	2.4	bone marrow	4	64	256	4	512	512	1024	0.5	2	Kiskunfélegyháza	Dél-Alföld
Salmonella sp. (Typhimurium)	2.48	bone marrow	2	32	128	4	256	256	1024	0.25	4	Daruszentmiklós	Közép-Dunántúl
**MIC_{50}**			**4**	**32**	**128**	**4**	**512**	**256**	**1024**	**0.03**	**1**		
**MIC_{90}**			**256**	**64**	**256**	**8**	**1024**	**512**	**1024**	**2**	**8**		

AMX – amoxicillin; AMC – amoxicillin-clavulanic acid (2:1 ratio); NEO – neomycin; SPE – spectinomycin; FLO – florfenicol; TIL – tilozin; TIA – tiamulin; LIN – lincomycin; ENR – enrofloxacin; COL – colistin; MIN – minocycline

Table S5. Statistical analysis of the resistance profiles of samples derived from ducks and geese, comparing the resistance levels of the two animal species for each antibiotic agent.

Antibiotics	Chi-square value	p-value	Degrees of freedom
Azithromycin	0.919415	0.63147	2
Cefotaxime	0.434481	0.50980	1
Ceftiofur	0.621527	0.73289	2
Ceftriaxone	3.310459	0.19105	2
Ciprofloxacin	0.905311	0.63594	2
Doxycycline	1.299939	0.52206	2
Imipenem	2.545125	0.28011	2
Chloramphenicol	0	1	1
Levofloxacin	2.603166	0.27210	2
Potential sulfonamide*	0	1	1
Amoxicillin-clavulanic acid**	0	1	1
Minocycline	1.65610	0.43690	2

*trimethoprim – sulfamethoxazole 1:19 ratio; **2:1 ratio
